# Untargeted metabolomic profiling and genome mining of endophytic *Stenotrophomonas maltophilia* strain 3A reveal a rich source of bioactive secondary metabolites

**DOI:** 10.3389/fmicb.2026.1792452

**Published:** 2026-05-12

**Authors:** Pramod Kumar Sahu, Krishna Nanda Dhal, Nakul Kale, Amrita Gupta, Nidhi Rai, Deepak Singla, Alok Kumar Srivastava

**Affiliations:** ICAR-National Bureau of Agriculturally Important Microorganisms, Maunath Bhanjan, Uttar Pradesh, India

**Keywords:** biosynthetic gene clusters, endophyte, LC-MS/MS, plant-microbe interaction, secondary metabolites, *Stenotrophomonas maltophilia*, untargeted metabolomics

## Abstract

The changing climate has posed several constraints in agricultural productivity. The losses from the diseases like bacterial wilt caused by *Ralstonia solanacearum* are a major threat to tomato cultivation, however, the management is still difficult. As a sustainable management practice, the endophytic bacterial strains are emerging as potential biocontrol agents. However, the mechanisms of action are poorly understood. Thus, in the present study, we assembled and evaluated bacterial endophyte *Stenotrophomonas maltophilia* 3A for antagonism and their underlying mechanism using multi-omics approach. This strain exhibited strong antagonistic activity against *R. solanacearum* along with having good colonization potential. The FTIR and LC-MS/MS-based analysis revealed that *S. maltophilia* 3A produced a diverse array of metabolites having potential as biocontrol agent. Pathway enrichment analysis using KEGG revealed significant involvement of glycerophospholipid biosynthesis, indole alkaloid biosynthesis, diterpenoid biosynthesis, and the phenylpropanoid pathway. Confocal laser scanning microscopy (CLSM) confirmed effective colonization of tomato root and stem tissues of *S. maltophilia* 3A, with strong localization observed in cortical and vascular regions, indicating a stable endophytic relationship. The presence of compounds like alkaloids, phenolic acids and antimicrobial compounds indicate the ability of the endophyte for a successful colonization in the plants. The results of LC-MS/MS were further confirmed by the whole genome analysis of *S. maltophilia* 3A followed by study of biosynthetic gene clusters (BGCs) using antiSMASH analysis. The whole genome analysis showed that the five BCGs present in 3A biosynthesize the secondary metabolites and bioactive peptides responsible for plant growth and health promotion. The results of this study indicated potential mechanisms of endophyte *S. maltophilia* 3A for biocontrol of plant pathogens. This could be fundamental in deciphering the functional roles of this endophyte in improving plant health.

## 1 Introduction

Endophytes are microbes living inside the plant tissues and are not having any harm to its host plants (Sahu et al., 2023). With advent of holobiome concept, it is well accepted that the associated microbial symbionts of the plants have very crucial effect on plant physiology and development, especially in the fitness against biotic and abiotic stresses (Phurailatpam et al., 2024). The metabolites produced by the endophytes are largely being explored for plant protection measures against several pathogens (Elshahawy and Haggag, 2026). These benefits comprise the potential for secondary metabolites with agronomic and pharmaceutical significance.

The biological control is an effective and environmentally beneficial way of reducing the use of agrochemicals. The production of bioactive compounds is one of the most crucial processes through which biocontrol agents protect plants, particularly species of agricultural significance (Rojas López-Menchero et al., 2026). Alkaloids, flavonoids, phenolics, non-protein amino acids, glucosinolates, cyanogenic glycosides, and terpenoids constitute significant secondary metabolites that safeguard plants (El-Shatoury and Diab, 2026). Endophytes have the ability to generate a wide range of bioactive compounds which function in chemical defence of plants due to their long-term interaction with associated microbes, and defence of their niche against pathogen species which damage the ecological surroundings (Mageshwaran et al., 2022).

Existing endophytic bacterial metabolomic investigations have mostly focused on well-known species like *Bacillus, Pseudomonas*, and *Streptomyces*. *Stenotrophomonas maltophilia* has been frequently neglected despite its increasing prevalence in endosphere microbiota databases (Sahu et al., 2019). In cases where studies on its metabolome are conducted, they are typically constrained by a lack of sophisticated quantitative methods, low-resolution analytical methods, or limited chemical detection ranges. As a result, little is known about *S. maltophilia’s* entire metabolic reserve and ecological significance at the metabolomic level, especially with reference to plant colonization, stress mitigation, and plant-microbe interactions. High-throughput metabolomics is a potent tool for clarifying the intricate metabolic profiles of microbial endophytes, especially when used in an untargeted way with liquid chromatography–mass spectrometry (LC-MS/MS) (Sahu et al., 2025). However, there are very smaller number of studies that utilized untargeted LC-MS/MS approach for study of endophytes like *S. maltophilia* with whole genome analysis for agriculturally important traits.

Thus, the present study conducted with a comprehensive untargeted LC-MS/MS-based metabolomics study of *S. maltophilia* strain 3A in combination of whole genome sequencing. We studied the biosynthetic gene clusters of the *S. maltophilia* strain 3A whole genome using antiSMASH analysis, which helps identify functionally involved gene clusters of secondary metabolites and bioactive antimicrobial peptides. This analysis enables us to further understand the growth-promoting and antagonistic mechanisms of the endophyte at the genomic level.

This study adds distinctive knowledge into the metabolic flexibility of endophytic *S. maltophilia* strain 3A and proves the efficacy of comprehensive metabolomic profiling for discovering functionally relevant biochemical compounds. By addressing a critical gap in the metabolomic characterisation of this species, our findings integrate a baseline for future investigations to fully harness the biotechnological potential of *S. maltophilia* strain 3A.

## 2 Materials and methods

### 2.1 Culture preparation

The endophyte *Stenotrophomonas maltophilia* strain 3A (Accession No. NAIMCC-B-02083 at National Agriculturally Important Microbial Culture Collection, India) was isolated in our earlier study from the Tomato (*Solanum lycopersicum* L.) plants grown in Eastern Indo-Gangetic plains, exhibiting no disease symptoms. The pure colonies preserved in glycerol stock were revived in nutrient agar medium for further use.

### 2.2 Pathogen culture and *in vitro* antagonism assay

Different virulent strain of *Ralstonia solanacearum* was collected from infected tomato plants and cultured on triphenyl tetrazolium chloride (TTC) agar at 28 °C. For antagonism assays, dual-culture confrontation tests were performed on nutrient agar plates. A 5 μL suspension of *S. maltophilia* (OD₆₀₀ = 0.5) was spot-inoculated 2.5 cm from a streak of *R. solanacearum*. Plates were incubated at 28 °C for 48 h (Messiha et al., 2007). Inhibition zones were measured in millimeters. The experiment was performed in triplicate. Isolates were placed in sterile 2 mL centrifuge tubes and inoculated with nutritional broth (NB) for 24 h at 37 ± 1 °C. These cells are then centrifuged to obtain the supernatant, which serves as the extracellular compounds, while the pellet, which serves as the intracellular compounds, is resuspended in 1x phosphate buffer solution and tested against *R. solanacearum* swabbed on TTC agar plate to determine which compound exhibits this antagonistic activity by the well diffusion method.

### 2.3 Study of nature of antimicrobial compound by *in vitro* antagonism assay

From the 2-day old culture of *S. maltophilia* 3A, three types of fractions were taken, one with whole live cells, one bead-beaten cell-free extract, and one cell-free extract after sonication. These three fractions were tested for the suppression of *Ralstonia solanacearum* and production of excessive exopolysaccharides which is directly related to the virulence of this pathogen.

Further, the bead-beaten cell free extract was tested for their concentration depended effects on the *Ralstonia solanacearum* colony fluidity (production of excessive exopolysaccharides) which is directly related to the virulence of this pathogen. This cell free extract was used in four different concentrations, 0.5x, 1x, 5x, and 10x. The data was recorded after 1 day, 2 days, and 3 days.

### 2.4 Genome sequencing, assembly, and analysis

Genomic DNA was extracted using the NucleoSpin microbial DNA extraction kit (Macherey-Nagel, Germany) as per the manufacturer’s protocoland sequenced on Illumina NovaSeq platform with paired-end read of length 2 × 151 bp. The quality control of raw data was performed using Falco and fastP software using polyX trimming, with low complexity filter and correction of read on overlap region (de Sena Brandine and Smith, 2021). The high-quality paired-end reads were assembled using Unicycler software. The quality of assembled genome was assessed using QUAST and BUSCO software and the final assembly was subjected to gene prediction using Bakta software (Gurevich et al., 2013; Manni et al., 2021). The pathway analysis was performed using BlastKOALA webserver on KEGG database (Kanehisa et al., 2016).

### 2.5 Detection of secondary metabolite biosynthetic gene clusters (SMBGC)

antiSMASH (Antibiotics and Secondary Metabolites Analysis Shell) webserver version 8.0.2 (Blin et al., 2025) was used to detect the secondary metabolite Biosynthesis Gene Clusters (SMBGCs) in the assembled genome. The genome assembly fasta file was provided as input, and the webserver first annotate the assembly using Prodigal and thereafter predict SMBGCs in the genome. BAKTA software annotated genbank file was used as input for prediction of SMBGC. The core structures of antibiotics and secondary metabolites gene clusters were identified and compared with known gene clusters of other species.

### 2.6 Molecular identification

The 16S rRNA gene was amplified using universal bacterial primers 27F (5’-AGAGTTTGATCMTGGCTCAG-3′) and 1492R (5’-TACGGYTACCTTGTTACGACTT-3′). PCR conditions included an initial denaturation at 95 °C for 5 min, followed by 35 cycles of denaturation (95 °C, 30 s), annealing (55 °C, 30 s), and extension (72 °C, 90 s), with a final extension at 72 °C for 10 min. Amplicons were sequenced commercially, and the resulting sequence was subjected to BLAST analysis using Ez-Biocloud NCBI database. The closest match was identified and submitted to the NCBI database. The phylogenetic relatedness of the isolates was assessed by the Neighbour-Joining method using MEGA11.0 (Saitou and Nei, 1987; Tamura et al., 2021). Additionally, the whole genome assembly was also subjected to taxonomic assignment using GTDB-tk toolkit. Gtdbtk classify_wf command was used for whole genome based taxonomic classification (Chaumeil et al., 2019).

### 2.7 *In situ* visualization of endophytic colonization using CLSM

To assess colonization and viability of *S. maltophilia* on tomato roots, dual staining with SYTO 9 was performed. Tomato seedlings were inoculated with the bacterial suspension and incubated under sterile conditions for 5–7 days. Root segments were gently washed with phosphate-buffered saline (PBS) to remove loosely attached cells and then stained using the LIVE/DEAD^™^ BacLight^™^ Bacterial Viability Kit (Invitrogen), which includes SYTO 9 (Sahu et al., 2021). The staining solution was prepared according to the manufacturer’s instructions and applied to the root and samples were incubated for 15 min in the dark. SYTO 9 stained live bacterial cells green (excitation 480 nm, emission 500 nm). Stained roots were mounted on glass slides and imaged using a confocal laser scanning microscope (Nikon A1R HD25). Z-stack and merged images were captured to visualize the spatial distribution and viability of bacterial cells on root surfaces.

### 2.8 Crude metabolite extraction

The crude extracts of potential bacterial endophyte *S. maltophilia* 3A showed antagonistic properties. Briefly, the endophytic isolate was aseptically inoculated in nutrient broth medium (125 mL), and the flask was incubated at 30 °C with 150 rpm continuous shaking (REMI CIS-24 PLUS incubator shaker). For extraction, HPLC grade solvents are used. After one week incubation, the culture broth was mixed with n-hexane in a ratio of 1: 1 (v/v). This mixture was added to a separating funnel and shaken vigorously for 10 min. The mixture was allowed to settle, and each phase was settled and collected separately in a fresh collector. The above same steps were repeated with chloroform solution. Ultimately, the collected extract was air-dried in the dark and gently scraped. The dry extract was stored at 4 °C in an amber colour tube for further analysis (Sahu et al., 2025).

### 2.9 Fourier-transform infrared (FTIR) spectroscopy of crude biomass extract

Fourier-transform infrared (FTIR) spectroscopy was employed to analyze the functional groups present in the crude extract obtained from *S. maltophilia*. After Hexane-chloroform extraction and solvent evaporation, the resulting dry biomass 2 mg was finely ground with spectroscopic-grade potassium bromide (KBr) 200 mg and compressed into a transparent pellet using a hydraulic press under 10 tons of pressure (Ojeda and Dittrich, 2012). The pellet was loaded onto the iD1 head and the percentage transmittance was recorded using Omnic Paradigm software (Thermo Scientific, USA). The peaks corresponding to molecular vibrations were recorded and compared in the sample in response to infrared exposure.

### 2.10 Metabolite profiling using LC-HRMS analysis

#### 2.10.1 Sample preparation for LC-HRMS

For the LC-HRMS study, the sample was prepared by mixing 100 mg of *S. maltophilia* 3A crude extract in 1.5 mL of solvent (methanol and HPLC-grade water in the ratio of 80:20) and homogenized at 750 rpm for 30 min using an Eppendorf Thermo-mixer C (Eppendorf, Hamburg, Germany) at 25 °C. The mixture was then centrifuged at 3500 rpm for 10 min at 25 °C. The supernatant was filtered using a 0.22 μm syringe filter. From this, a 5 μL sample was injected into the C18 RP-HPLC column (Hypersil GOLD™, Waltham, MA, United States: particle size 1.9 μm × 2.1 mm × 100 mm). The reversed-phase chromatographic separation was performed in a gradient of solutions ranging from 0% ethanol to 95% ethanol phase in 0.1% formic acid (Hou et al., 2021).

#### 2.10.2 LC-HRMS data acquisition

The three technical replicates of the bacterial endophyte *S. maltophilia* 3A crude extract were used for total metabolomics analysis. A Thermo Scientific Tribrid High-Resolution Accurate Mass Spectrometer “Orbitrap Eclipse” (Sunnyvale, CA, United States), coupled with an Ultra-High-Pressure Liquid Chromatography system (DionexUltiMate 3,000 RSLC; Sunnyvale, CA, United States) and a Heated Electrospray Ionization (HESI) source, was employed for sample analysis following chromatographic separation. The Orbitrap analyzer operated at a resolution of 60,000 in both positive and negative polarity modes, covering a mass range of m/z 100–1,000. The instrument settings included a 35% RF lens, a 25% normalized AGC target, and an intensity threshold of 2.0 × 105 for MS-OT (Master Scan). For ddMS2 OT HCD analysis, the parameters were configured as follows: quadrupole isolation mode with an isolation window of 1.5 m/z, HCD as the activation type, collision energies of 30, 45, and 60%, and an Orbitrap resolution of 15,000. We also set the normalized AGC target to 20% (Sahu et al., 2025).

#### 2.10.3 Data analysis

The mass analyzer’s raw data were processed using the “Compound Discoverer 3.3.2.31” workflow, which identifies differences between samples, predicts elemental compositions, fills gaps, and hides chemical background noise. The databases mzCloud (https://www.mzcloud.org/; accessed on 5 April 2024) and ChemSpider (https://www.chemspider.com/; accessed on 5 April 2024) were used to determine the compounds, and similarity searches were performed. We applied the mzLogic algorithm to rank the results from ChemSpider in order. Pre-processed data were normalized by total ion current (TIC) and log-transformed.

### 2.11 Biological pathway enrichment analysis

Metabolite annotation and pathway mapping were carried out using established metabolomics databases and analytical tools. Initially, batch normalization was performed based on quality control samples to ensure data consistency. Differential analysis of metabolites was conducted using both t-test and ANOVA to identify significantly varying compounds. Putative identification of metabolites was achieved by matching mass-to-charge (m/z) ratios with entries in the Metabolomics Workbench database (https://www.metabolomicsworkbench.org; accessed on 10 April 2024), resulting in the detection of 1,612 compounds. Subsequently, pathway analysis was performed using the pathway analysis module in MetaboAnalyst 5.0. The identified metabolites were uploaded into the platform and mapped to biological pathways using the KEGG database as a reference. Pathway enrichment and topology analyses were conducted by selecting the “General Metabolism” and “Secondary Metabolite Biosynthesis” pathway libraries to interpret the biological significance of the metabolomic profiles.

## 3 Results

### 3.1 Antagonistic activity against different strains of *Ralstonia solanacearum*

The antagonistic potential of strain 3A was further validated against nine isolates of *R. solanacearum* using both dual-culture and well diffusion assays (Supplementary Table S1). The highest inhibition rate was observed against strain RS3, with inhibition of 20 mm. Other strains such as RS1, RS2, and RS9 showed inhibition values of 22 mm, 19 mm, and 18 mm, respectively. The lowest inhibitory effect was observed against strain RS8 11 mm, indicating differential susceptibility among the *R. solanacearum* isolates.

The extracellular (supernatant) and intracellular (pellet) extracts of strain 3A also exhibited inhibitory zones on TTC agar plates, confirming that bioactive metabolites produced by the bacteria are responsible for the observed antagonism.

### 3.2 Study of nature of antimicrobial compound by *in vitro* antagonism assay

**Figure 1:**
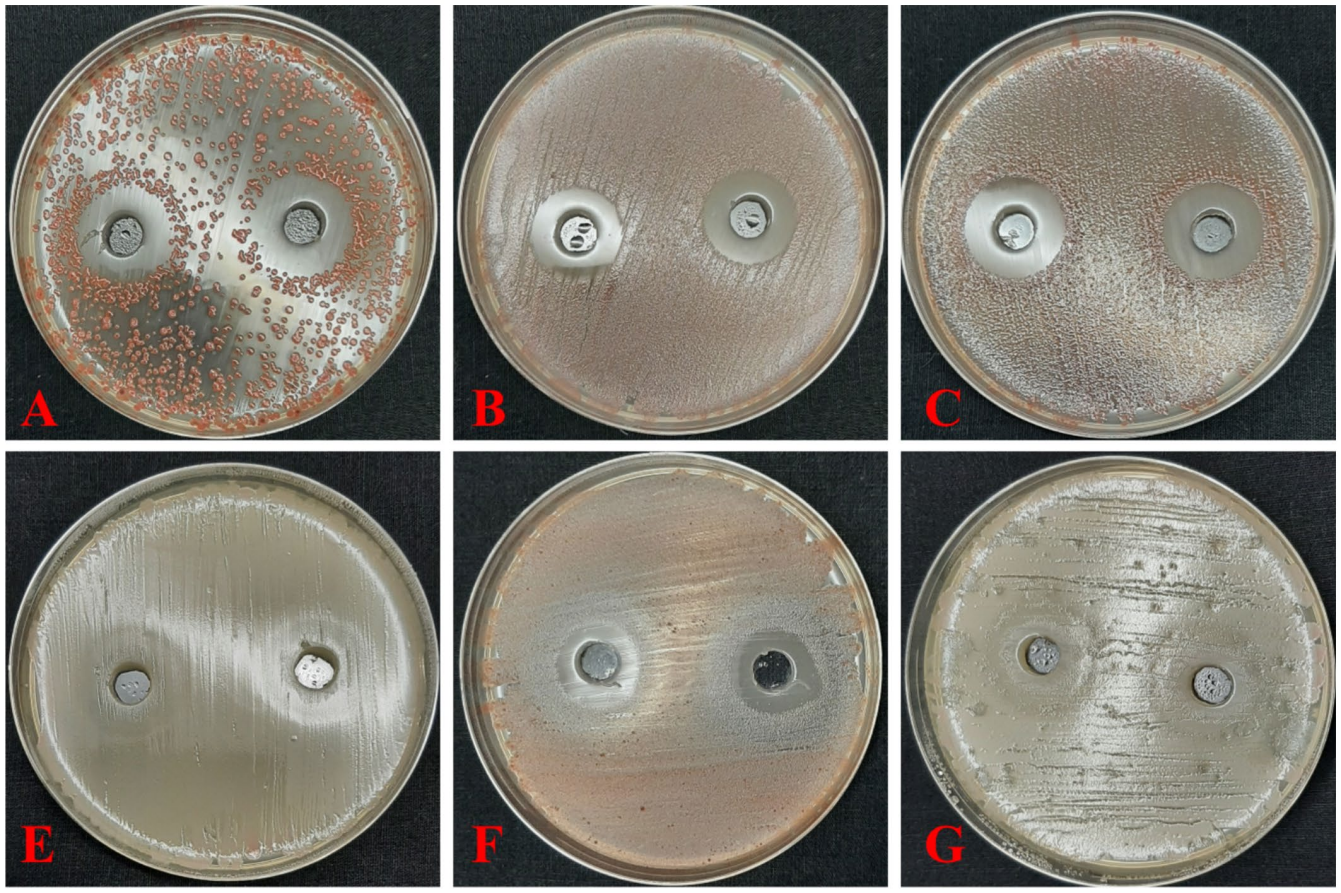
Well diffusion assay showing inhibition zones produced by metabolites of *Stenotrophomonas maltophilia* 3A against different *Ralstonia solanacearum* strains; (**A–G** represent 6 different *R. solanacearum* strains). Clear zones around wells indicate suppression of bacteria growth, demonstrating the antagonistic potential of the bacterial isolate.

**Figure 2:**
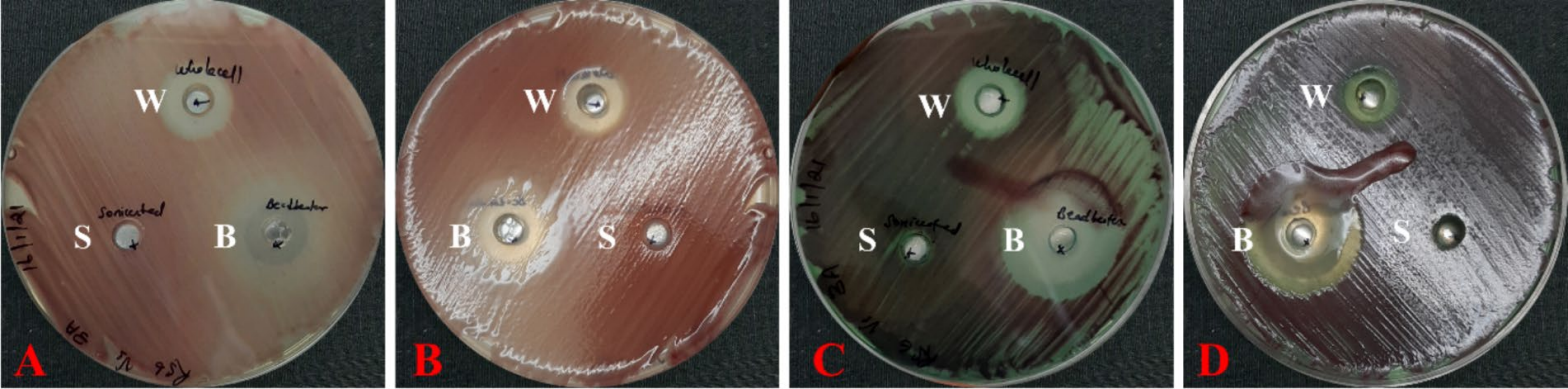
Nature of different cell fractions in *Stenotrophomonas maltophilia* 3A and its bacteriostatic nature: **(A)** Reverse side of Petri plate after 2 days of incubation, **(B)** Front side of Petri plate after 2 days of incubation, **(C)** Reverse side of Petri plate after 7 days of incubation, and **(D)** Front side of Petri plate after 7 days of incubation. W = Whole live cells; B = Bead beaten cell-free extract; C = Cell-free extract after sonication.

**Figure 3:**
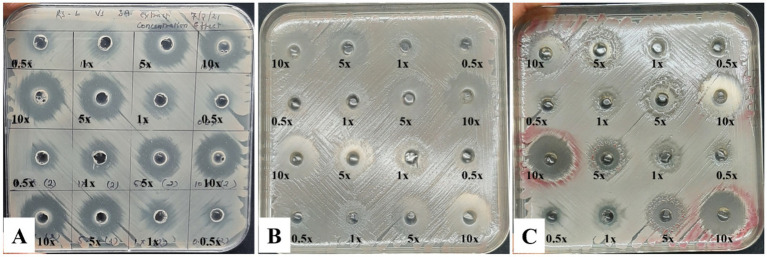
Effect of cell free extracts of *Stenotrophomonas maltophilia* 3A on *Ralstonia solanacearum*: **(A)** After 1 day, **(B)** after 2 days, and **(C)** after 3 days.

The *In vitro* antagonism study with *R. solanacearum* showed distinctive patterns (Figures 1–3). After 2 days of incubation whole cells and bead beaten fractions were having clear suppression zone (Figures 2A, B). However, the bead beaten fraction was getting another bigger zone of high exopolysaccharide producing cells above the suppression zone. In the case of sonicated fraction after 2 days, there was no suppression zone.

After 7 days, the whole cells fraction maintained the suppression zone and there was no appearance of high exopolysaccharide producing cells (Figures 2C,D). But in beat beaten fractions, the clear suppression zone was disappeared and the region was having high exopolysaccharide producing cells. Even in the bigger zone of high exopolysaccharide producing cells, the fluidity was enhanced and the exopolysaccharide was flown in the Petri plate. In the case of sonicated fraction after 7 days (Figures 2C,D), though there was no suppression zone, but the metabolites present in this section have suppressed the exopolysaccharide production.

In dose dependent experiment, this cell free extract was able to suppress *Ralstonia solanacearum* after 1 day of incubation and very clear suppression zone was found in all the concentrations (Figure 3). Two days onwards the appearance of fluidal colonies started from 0.5x and 1x zones. After three days, higher abundance of fluidal colonies seen from 0.5x,1x, and 5x zones. The 10x concentration of the *S. maltophilia* 3A cell free extract was able to prevent appearance of any fluidal type colony, indicating reach of a cidal concentration from the suppression zone.

### 3.3 Molecular identification and phylogenetic analysis

**Figure 4:**
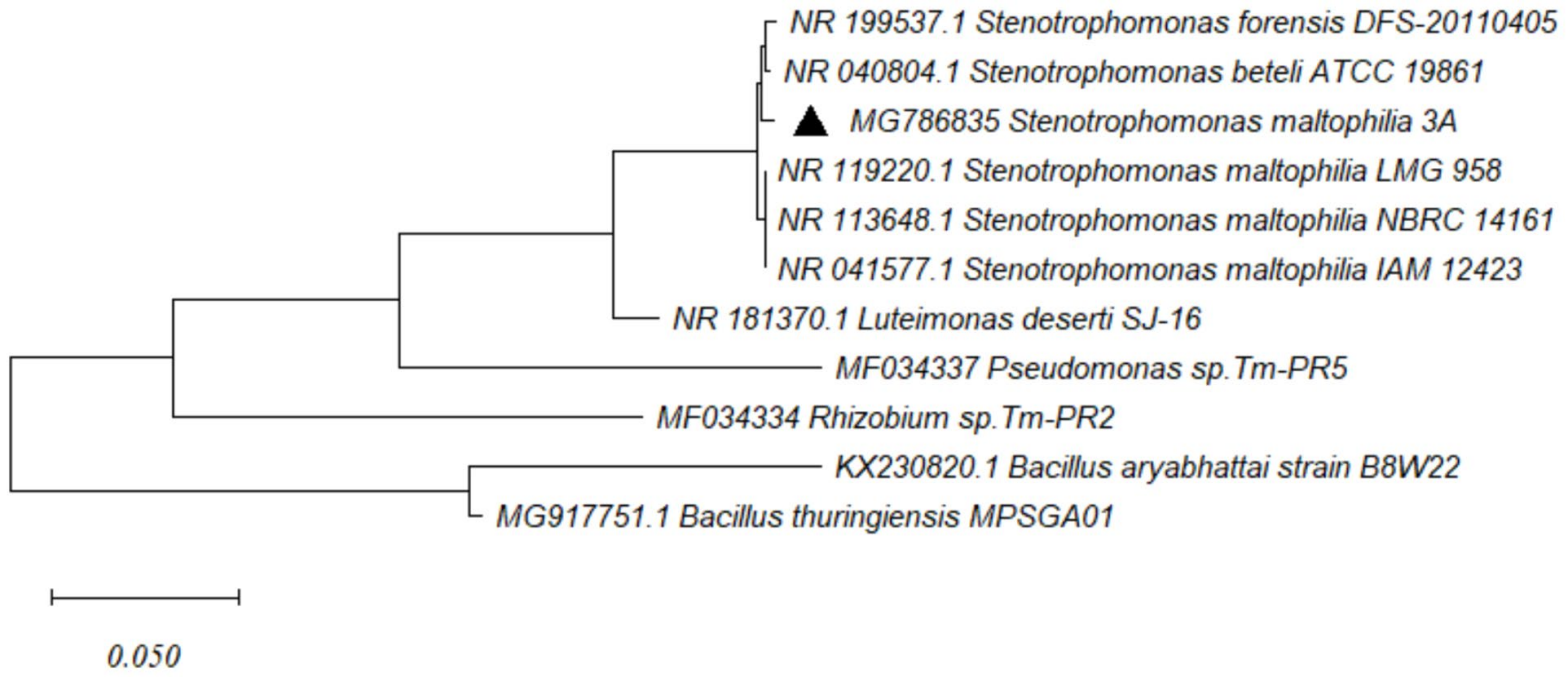
Phylogenetic tree based on *16S rRNA* gene sequences showing the relationship of isolate 3A with reference strains of *Stenotrophomonas maltophilia*. The tree was constructed using the Neighbor-Joining method in MEGA 11.0 with 1,000 bootstrap replications.

**Figure 5:**
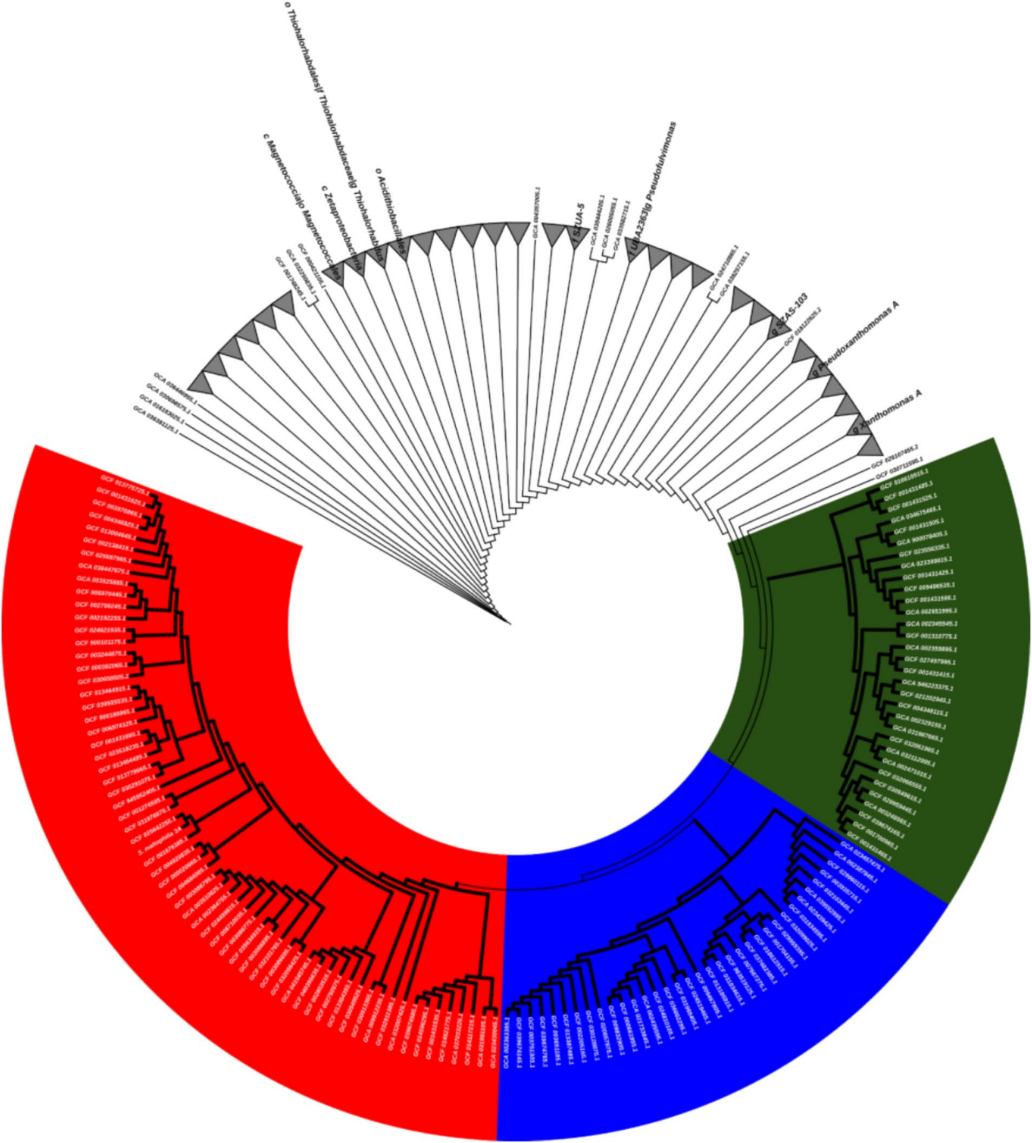
Phylogenetic tree constructed using GTDBtk based on whole gene sequences showing the relationship of isolate 3A with other strains of *Stenotrophomonas maltophilia*.

The selected bacterial isolate, designated as strain 3A, was subjected to 16S rRNA gene sequencing. The obtained sequence (~1.5 kb) showed 99.89% similarity with *Stenotrophomonas maltophilia* based on BLAST analysis against the NCBI GenBank database. A phylogenetic tree constructed using the Neighbour-Joining method in MEGA 11.0 placed strain 3A in a clade with reference strains of *S. maltophilia*, confirming its taxonomic identity (Figure 4). GTDB-tk analysis identified that *S. maltophilia* 3A is highly similar to the *S. maltophilia* strain SG.Y2 (Figure 5).

### 3.4 Genome analysis of *Stenotrophomonas maltophilia* 3A genome

**Figure 6:**
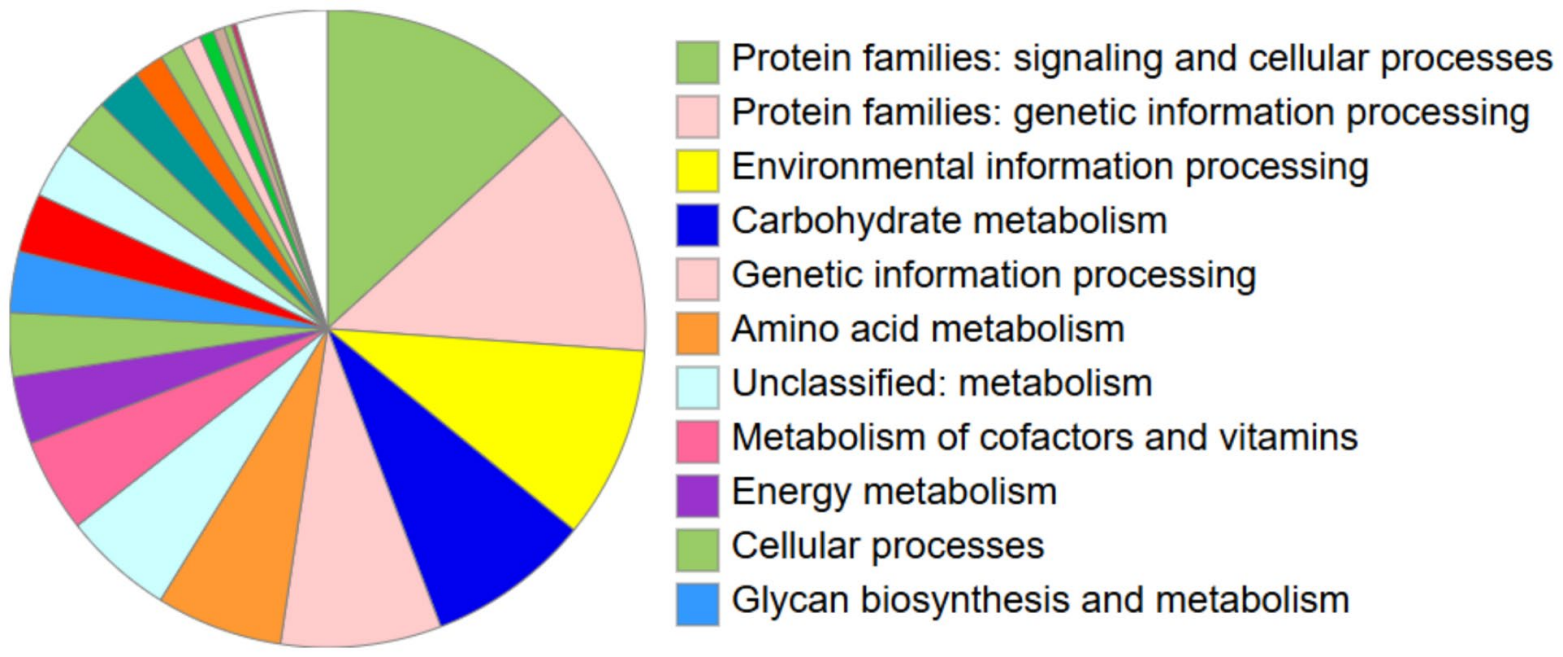
KEGG pathway analysis using BlastKOALA.

This study generates ~8.1 million high-quality pair-end reads from 8.20 million raw reads. The genome assembly of high-quality reads produces 35 contigs of which 21 contigs were more than 500 bp in length (Table 1). As evident from Table 1, the total genome assembly size of 4.09 Mb with largest contig of size 926,032 bp and N50 value of 734,238 was observed. BUSCO analysis showed 99.82% complete and only one missing gene showed a good quality of genome assembly. Furthermore, 3,638 protein coding genes along with 65 tRNA, 5 rRNA and 6 pseduogenes were predicted in the assembled genome of *S. maltophilia* 3A. KEGG pathway analysis has annotated 56.8% of genes into different functional categories (Figure 6).

**Table 1:** Data statistics of *Stenotrophomonas maltophilia* 3A genome.

Sr. no.	Particulars	Values
1	Raw reads (paired-end)	8,205,425
2	High-quality reads (paired-end)	8,109,120
3	No. of contigs	35
4	Contigs >500 bp	21
5	Largest contig size	926,032 bp
6	N50	734,238
BUSCO		
7	Complete genes	1,150 (99.82%)
8	Fragmented genes	1 (0.09%)
9	Missing genes	1 (0.09)
Genome annotation summary		
10	Protein coding genes	3,638
11	tRNA genes	65
12	rRNA genes	5
13	tmRNA	2
14	ncRNA	21
15	Pseudogenes	6
16	Hypothetical genes	132
17	OriC	1

### 3.5 Secondary metabolite biosynthetic gene clusters (SMBGCs) analysis

**Figure 7:**
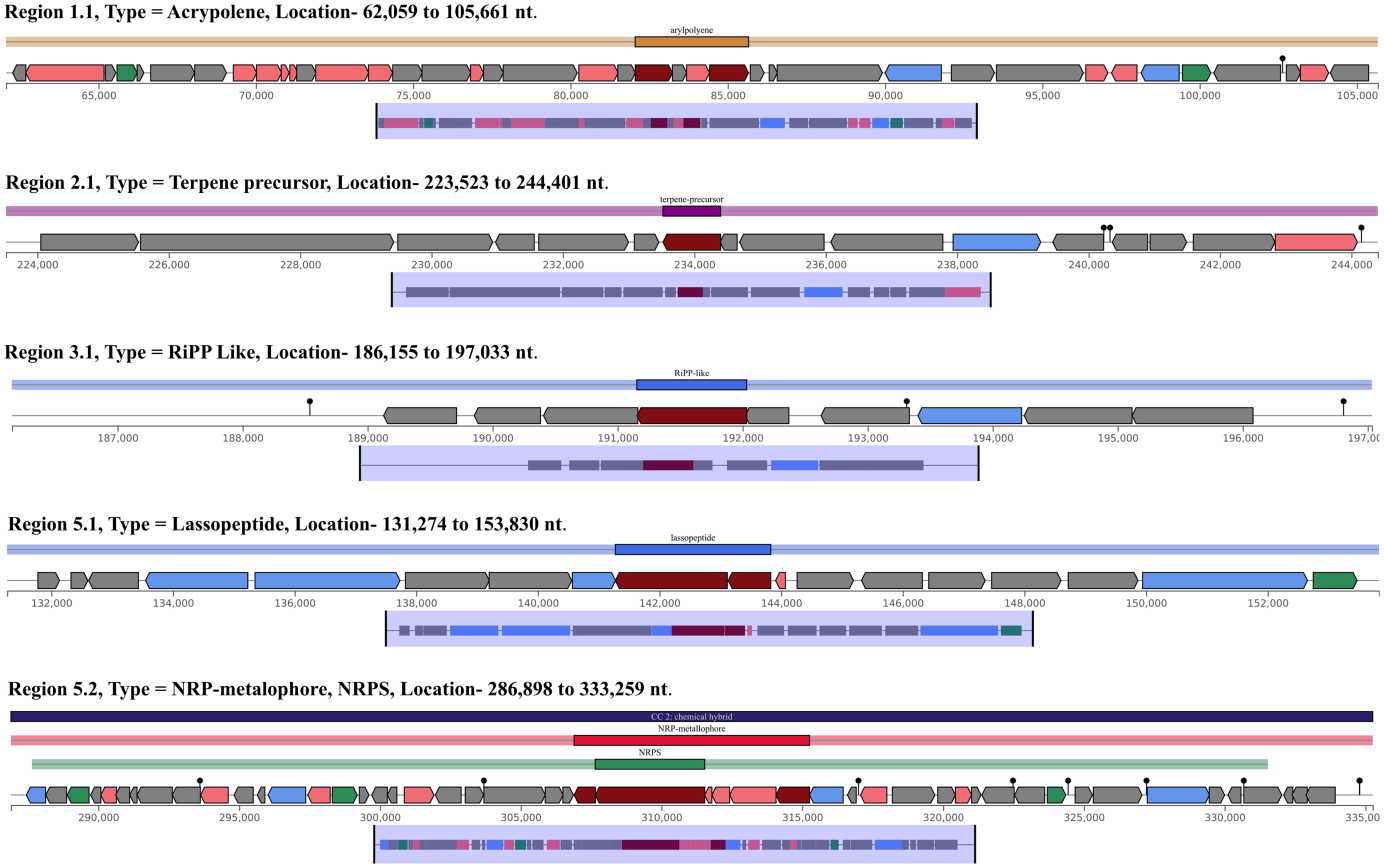
Proposed gene clusters of predicted secondary metabolites involved in the biosynthesis of various antibiotics in *Stenotrophomonas maltophilia* 3A genome, with related genes drawn in the different color (image produced by antiSMASH).

The secondary metabolite biosynthetic pathway analysis identified a total of five regions with different types of secondary metabolite biosynthetic gene clusters (BGCs), *viz.* arylpolyene, terpene-precursor, ribosomally synthesized and post-translationally modified peptides (RiPP) -like, lassopeptides and a hybrid of non-ribosomal peptide (NRP) -metallophore with NRP Synthetases (NRPS), as shown in Table 2 and Figure 7. When these BGCs were compared with known secondary metabolite gene clusters, the known cluster blast result for the region 1.1 type-arylpolyene, showed similarity with known homologous gene clusters involved in the biosynthesis of various antibiotics, including PKS, flexirubin (gene cluster from organism *Flavobacterium johnsoniae* UW101) and 3 others APEs from organism *Escherichia coli*CFT073, *Aliivibrio fischeri* ES114, etc., while the region 2.1 showed less similarities, potentially a novel analogue or derivative of Terpene-precursor, its blast result shows similarities with known clusters of astallatene, retigeranin/arathanatriene, (−)-ent-quiannulatene, boleracene, and some other unknown compounds. Similarly, region 3.1 showed very low similarity; it is possibly a new cluster of RiPPs and may be involved in the synthesis of novel products. Region 5.1 is Lassopeptides, which is also a ribosomally synthesized polypeptide, possibly involved in the synthesis of xanthomonin, sphingonodin, subterisin, caulonodin, siamycin, and other peptides. However, the known cluster blast result for the region 5.2 showed a hybrid cluster of NRP-metallophore and NRPS gene, which is most similar known homologous gene clusters of *S. maltophilia* K279a, involved in the biosynthesis of 9 NRPS (Type I), 2,3-dihydroxybenzoylserine, griseobactin, benarthin/dibenarthin, mirubactin, bacillibactin, myxochelin, paenibactin and 1 NRPS+PKS, myxochelin B/myxochelin N/myxochelin O/myxochelin P/myxochelin Q/myxochelin A. Together, these results signifies the multifaceted metabolic and antimicrobial potential of *S. maltophilia* 3A and support its role as a plant growth-promoting and antagonistic endophyte.

**Table 2:** Predictive gene clusters involved in biosynthesis of antimicrobials and secondary metabolites in *Stenotrophomonas maltophilia* 3A.

Region	From	To (nt.)	Size (kb)	No of genes	Type	Most similar biosynthetic gene clusters	MIBiG accessions	Similarity score	Gene cluster from organisms
1.1	62,059	105,661	43.6	39	Arylpolyene	APE Vf	BGC0000837	0.75	*Aliivibrio fischeri* ES114
2.1	223,523	244,401	20.9	16	Terpene-precursor	Astallatene	BGC0002397	0.47	*Arabidopsis thaliana*
3.1	186,155	197,033	10.9	9	RiPP-like	Hierridin B, Hierridin C	BGC0001962	0.27	*Cyanobium* sp. LEGE 06113
5.1	131,274	153,830	22.6	18	Lassopeptide	Xanthomonin II, Xanthomonin I	BGC0000580	0.51	*Xanthomonas gardneri* ATCC 19865
5.2	286,898	335,259	48.4	49	NRP-metallophore, NRPS	2,3-dihydroxybenzoylserine	BGC0002689	1.18	*Stenotrophomonas maltophilia* K279a

### 3.6 Root colonization visualized by confocal laser scanning microscopy (CLSM)

**Figure 8:**
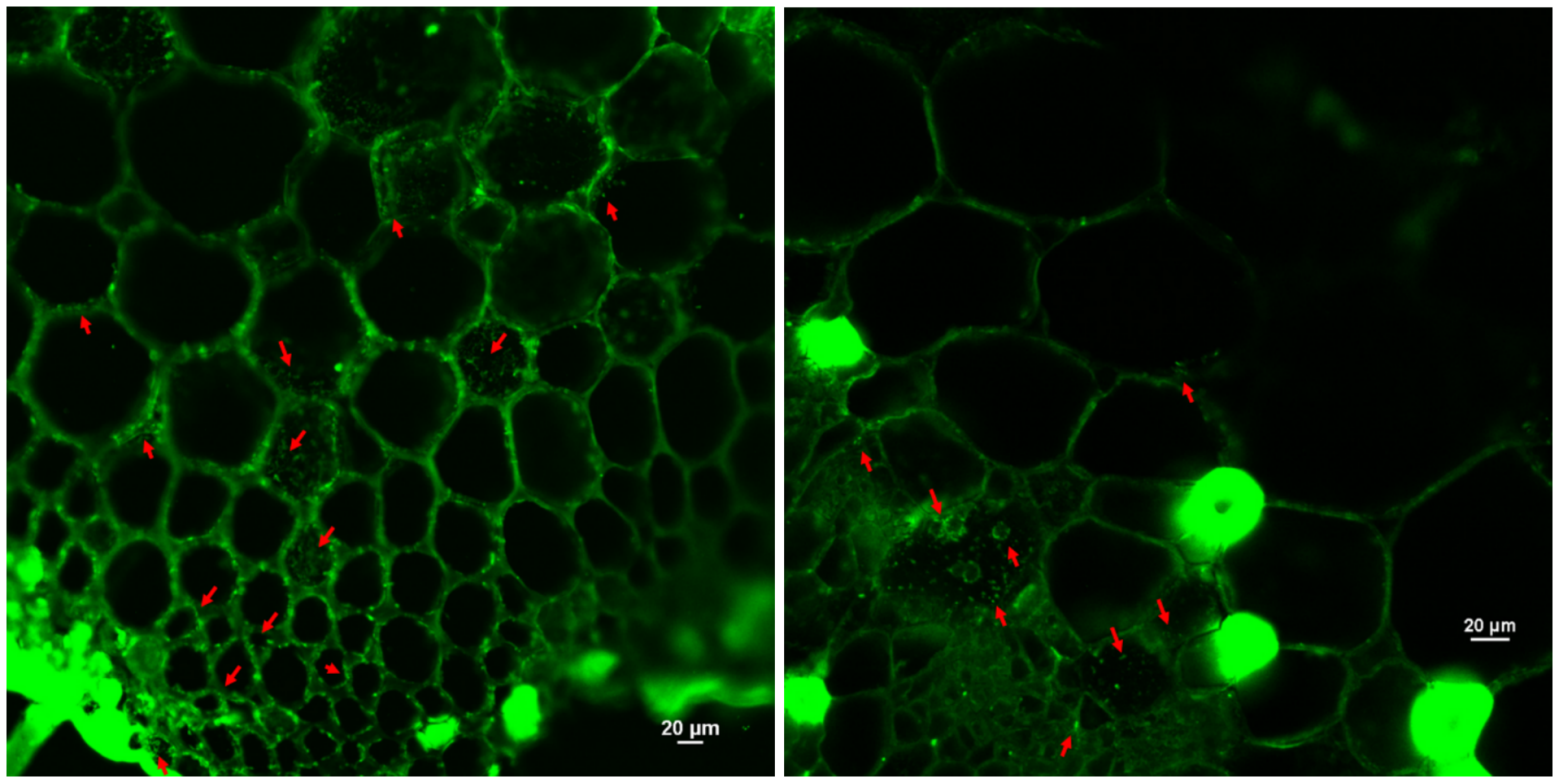
Confocal laser scanning microscopy (CLSM) images of tomato roots colonized by *Stenotrophomonas maltophilia* 3A. The 3A cells appear green (SYTO 9) and marked in red arrows. Images show colonization along the epidermis and intercellular regions (Scale bar = 20 μm).

CLSM imaging confirmed the colonization capability of *S. maltophilia* 3A on tomato roots. SYTO9 staining revealed extensive colonization with intense green fluorescence, indicative of live bacterial cells distributed along the root epidermis and in intercellular spaces. Colonization was visualized at depths of up to 20–30 μm within the root tissue (Figure 8).

### 3.7 Functional group identification by FTIR

**Figure 9:**
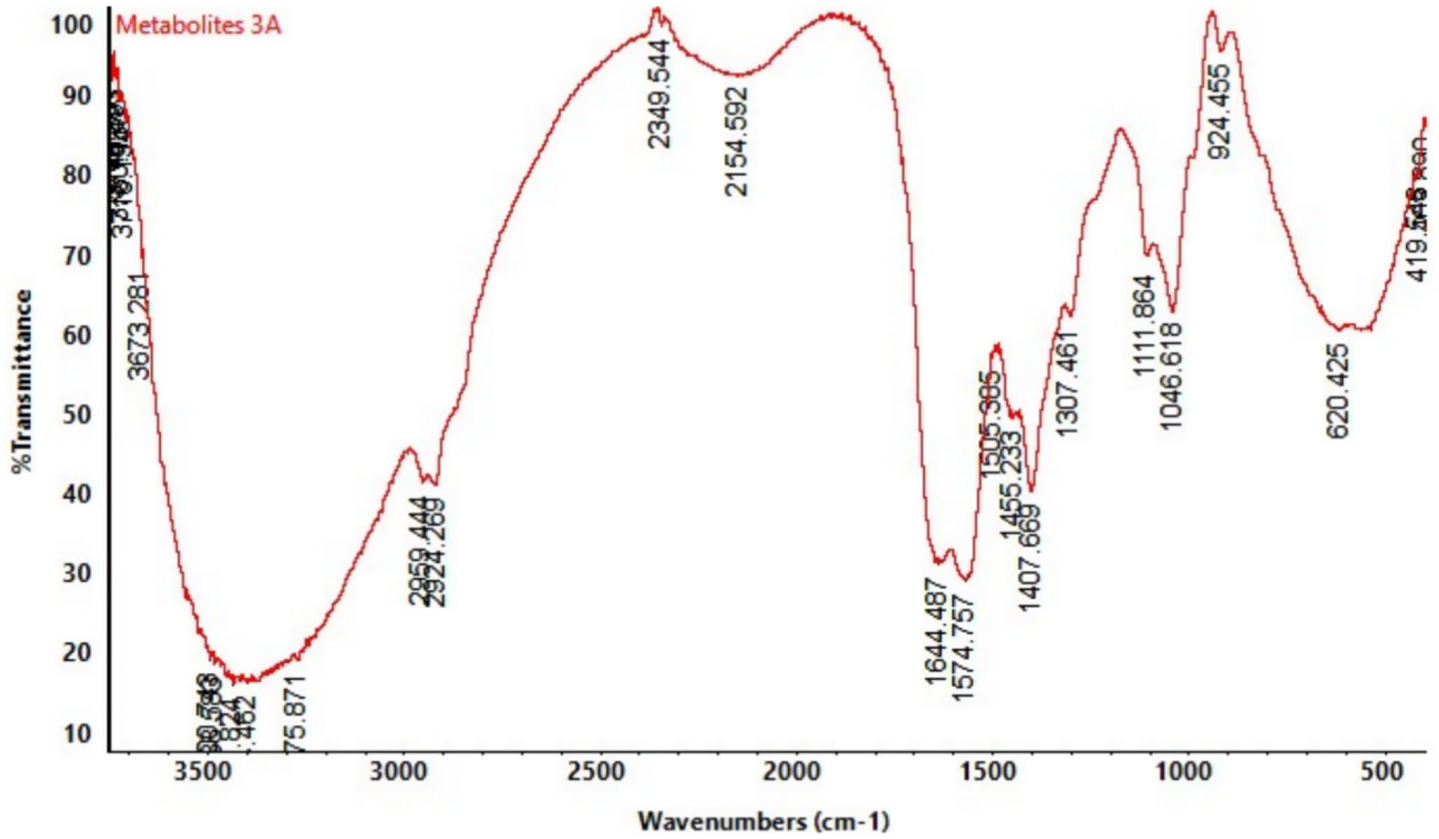
Fourier-transform infrared (FTIR) spectra showing distinct peaks corresponding to the antimicrobial compounds obtained from crude extract of *Stenotrophomonas maltophilia* 3A.

FTIR spectra of the crude hexane- chloroform extract of *S. maltophilia* 3A revealed multiple characteristic absorption peaks, suggesting the presence of diverse chemical groups. Key peaks corresponding to functional groups such as hydroxyls (3204.89 cm^−1^), carbonyls (1677.24 cm^−1^), phenolics (1409.72 cm^−1^), and ethers (1111.94 and 1047.08 cm^−1^), all linked to antimicrobial activity. Additional peaks for alkenes, aromatics, and halo compounds further indicate the presence of diverse bioactive metabolites that may contribute to the strain’s biocontrol potential, all of which are associated with antimicrobial properties (Figure 9).

### 3.8 Metabolomic profiling using LC-HRMS

**Figure 10:**
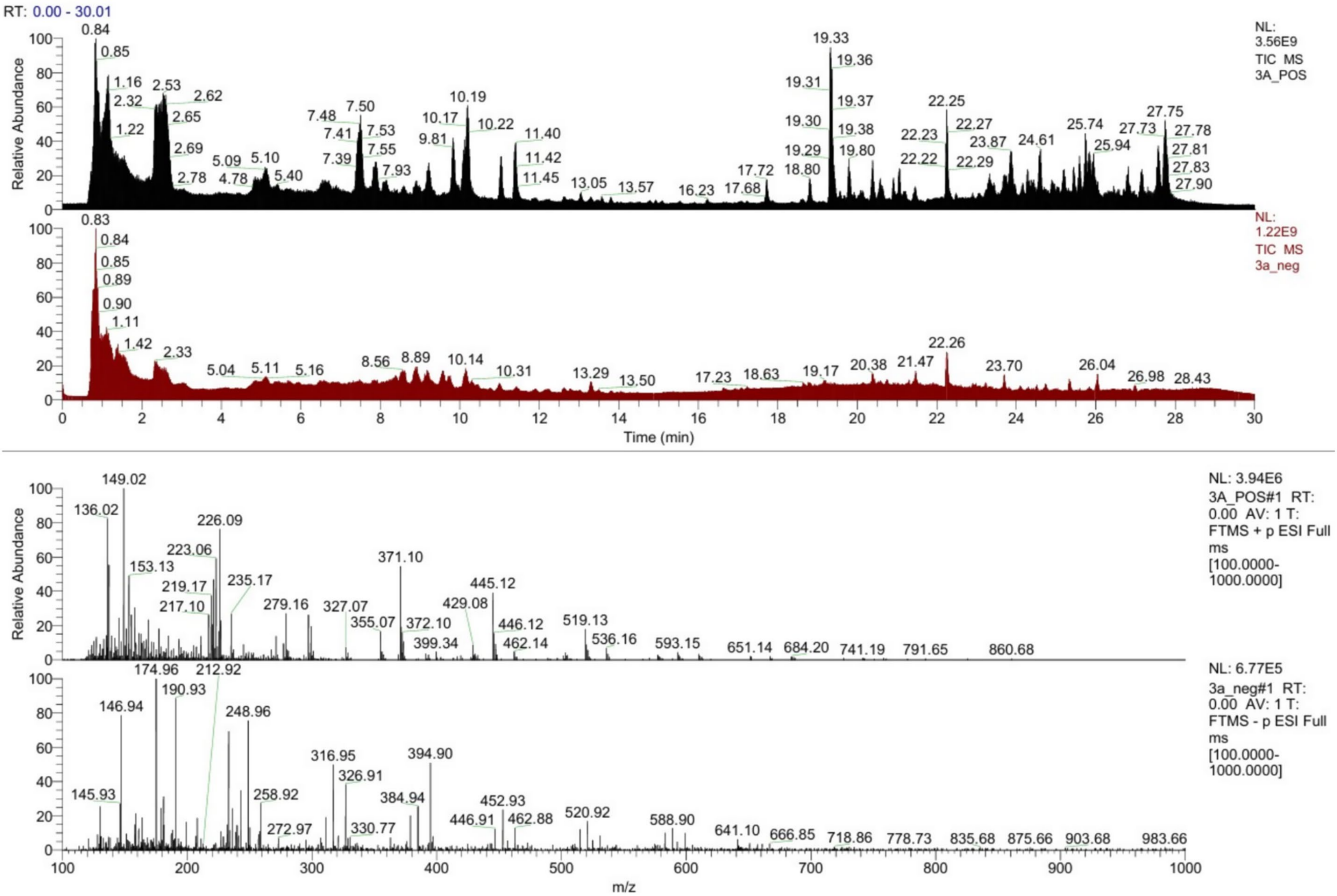
Total ion chromatogram (TIC) of the metabolites present in *Stenotrophomonas maltophilia* 3A crude extract.

A total of 1,612 metabolites were detected in the crude extract by LC-HRMS, as shown in Total Ion Chromatogram (TIC) (Figure 10). Metabolite identification revealed the presence of polyketides, alkaloids, fatty acids, and flavonoid-like compounds. Notably, secondary metabolites with known antifungal and antibacterial properties were identified, supporting the observed bioactivity (Supplementary Table S1).

**Figure 11:**
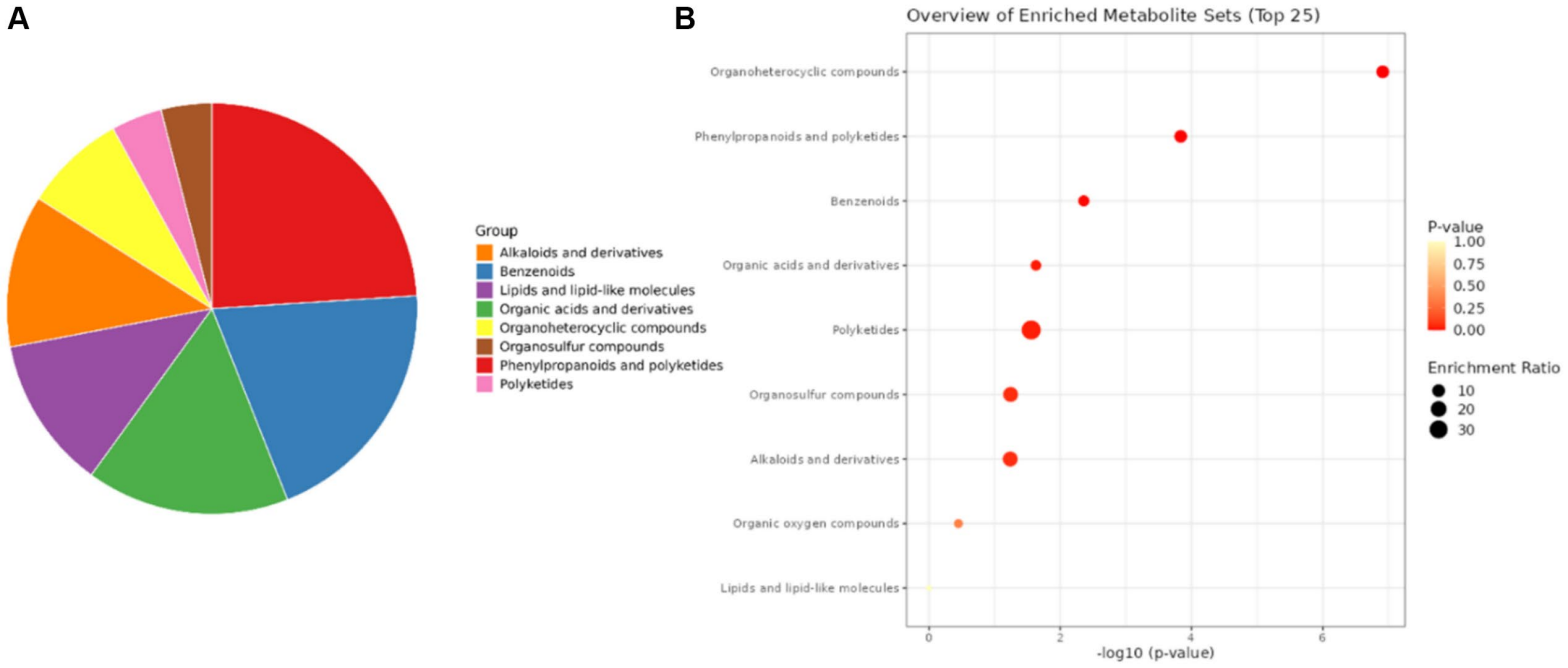
Classification and enrichment analysis of metabolites produced by *Stenotrophomonas maltophilia* 3A. **(A)** Pie chart illustrating the proportional distribution of identified metabolites into major chemical groups. **(B)** Dot plot summarizing the top enriched metabolite classes based on pathway-level enrichment analysis. The *x*-axis represents the −log₁₀ (*p*-value), reflecting the statistical significance of enrichment. Dot size indicates the enrichment ratio, while color gradient represents *p*-value intensity (from red = highly significant, to yellow = less significant).

Metabolomic profiling of *S. maltophilia* 3A revealed a diverse array of secondary metabolites spanning multiple chemical classes. Pie chart distribution showed that the most abundant metabolite classes were organonitrogen compounds, notably alkaloids and derivatives (24.3%), followed by organic acids and derivatives (18.2%), benzenoids (15.5%), lipids and lipid-like molecules (12.1%), and organo-heterocyclic compounds (10.3%). Other significant classes included phenylpropanoids and polyketides, organosulfur compounds, and polyketides, highlighting a broad biosynthetic capacity (Figure 11).

Dot plot analysis of enriched metabolite sets demonstrated that organonitrogen compounds (especially organo-heterocyclic compounds) and phenylpropanoids/polyketides were among the most significantly enriched pathways, with high enrichment ratios and low *p*-values (*p* < 0.001). Notably, benzenoid compounds, organic acids, and alkaloids also exhibited substantial enrichment, supporting their functional relevance. These results suggest that *S. maltophilia* 3A synthesizes a chemically diverse metabolome with strong representation of bioactive compounds potentially involved in antimicrobial activity, signalling, and plant-microbe interactions.

**Figure 12:**
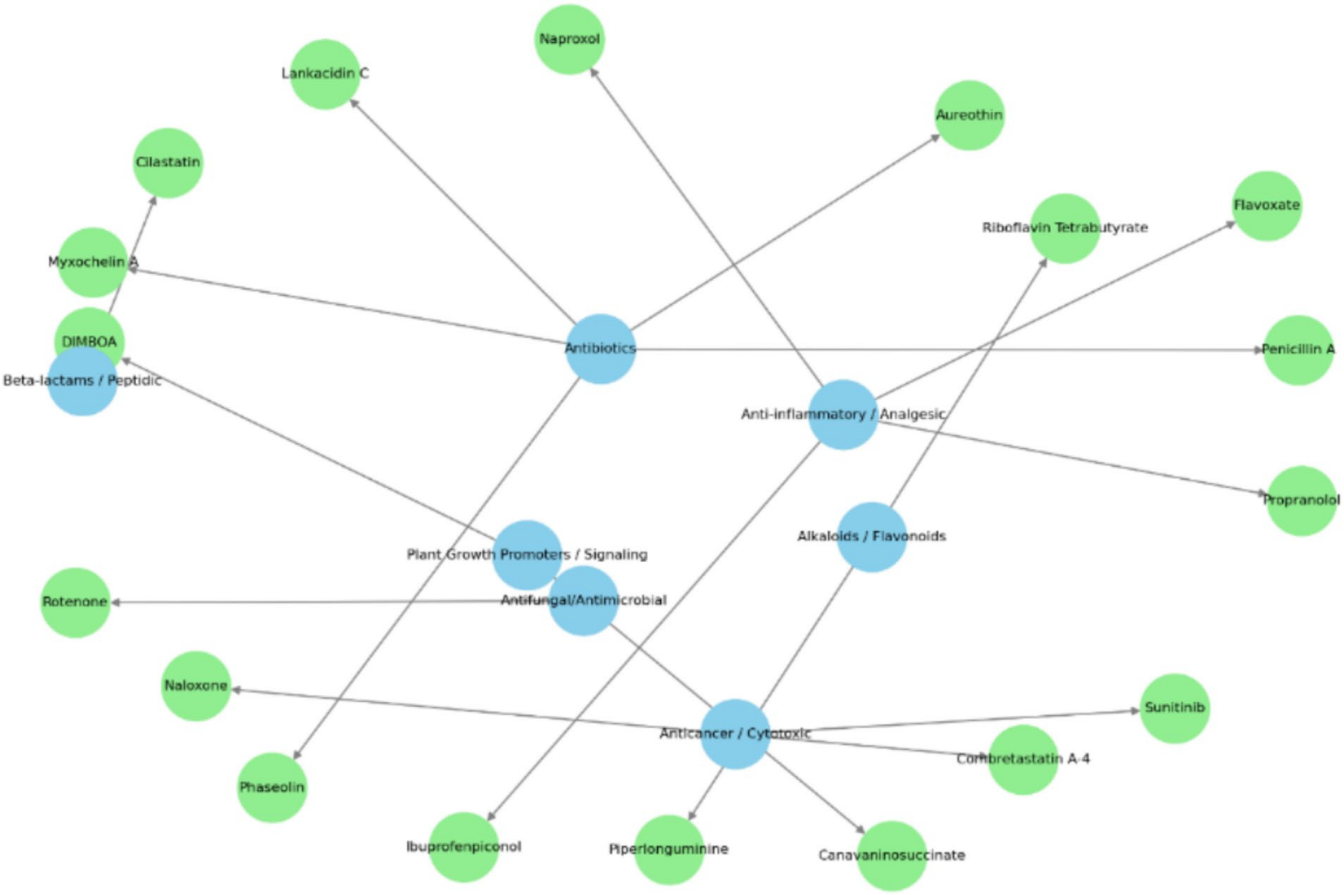
Functional network of bioactive metabolites identified in *Stenotrophomonas maltophilia* 3A.

**Figure 13:**
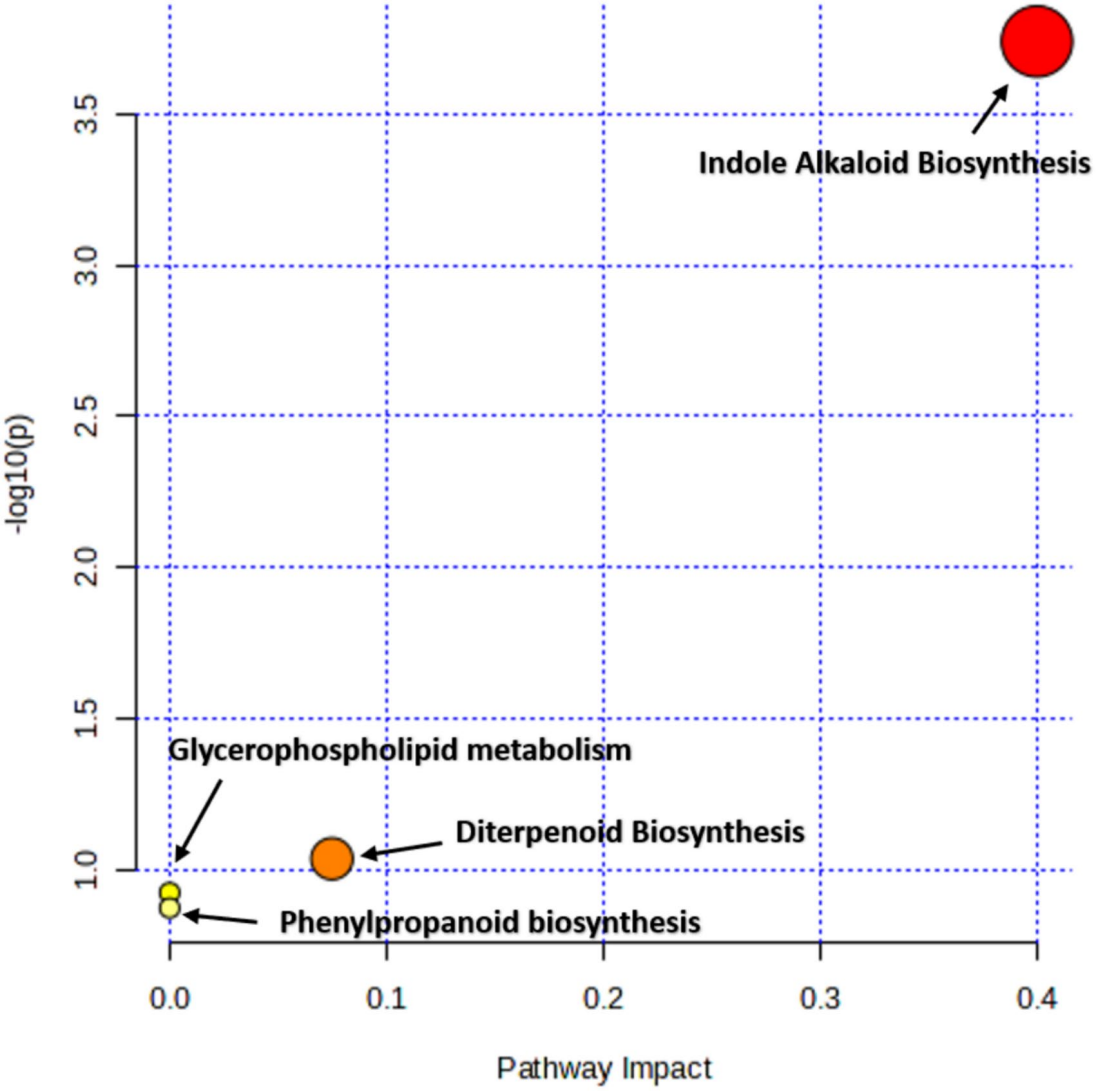
Pathway enrichment and impact analysis of metabolites produced by *Stenotrophomonas maltophilia* 3A using MetaboAnalyst 5.0. The bubble plot illustrates metabolite-associated pathways, with the *x*-axis representing pathway impact (based on topological analysis) and the *y*-axis showing –log₁₀ (*p*-value) for statistical significance.

The compound-function interaction network (Figure 12) highlights the diverse functional roles of metabolites identified from the crude extract of *S. maltophilia* 3A. Bioactive compounds (green nodes) were functionally categorized into core pharmacological and ecological roles (blue nodes), including antibiotic activity, anti-inflammatory/analgesic effects, plant growth promotion/signalling, alkaloid/flavonoid biosynthesis, and anticancer/cytotoxic potential. Notably, key antibiotic-related metabolites such as myxochelin, clavastatin, and rubratoxin tetra-acetate clustered around the “Antibiotic” node, indicating a strong potential for microbial antagonism. Similarly, compounds like canavaninosuccinate and piperlongumine were associated with the “Anticancer/Cytotoxic” class, suggesting therapeutic relevance. The node “Plant Growth Promoters / Signalling” was linked to metabolites such as DMBOA (2,4-dihydroxy-7-methoxy-1,4-benzoxazin-3-one), implicating this strain in plant-microbe interaction. Alkaloids and flavonoids, represented by compounds like flavoxate and penicillin A, formed another distinct cluster, indicating metabolic specialization. This integrative network underscores the multifunctionality of the *S. maltophilia* 3A metabolome and its potential utility in agriculture and biomedicine. Pathway enrichment and impact analysis of metabolites produced by *S. maltophilia* 3A using MetaboAnalyst 5.0 showed a bubble plot indicating a closure impact of Phenylpropanoid biosynthesis, glycerol phospholipid metabolism, and diterpenoid biosynthesis (Figure 13).

### 3.9 Biological pathway analysis

Metabolite annotation and KEGG-based pathway enrichment *via* MetaboAnalyst 5.0 highlighted significant contributions from glycerophospholipid biosynthesis, indole alkaloid biosynthesis, diterpenoid biosynthesis, and the phenylpropanoid pathway. Within the glycerophospholipid biosynthesis pathway (Supplementary Figure S1), we identified phosphoethanolamine (m/z 229.05, RT 14.928 min), suggesting active membrane lipid turnover and potential involvement in biofilm formation and host colonization. The presence of Vindoline (m/z 161.1073, RT 4.26 min) and 3 alpha strictosidine (m/z 176.0707, RT 5.73 min) in the indole alkaloid biosynthesis pathway (Supplementary Figure S2) confirmed the strain’s capacity for auxin biosynthesis, potentially enhancing root development and plant growth. In the diterpenoid biosynthesis pathway (Supplementary Figure S3), detection of Gibberellin A24 (m/z 449.2430, RT 8.91 min) suggests synthesis of compounds with known roles in antimicrobial defense. Furthermore, in phenylpropanoid pathway (Supplementary Figure S4) intermediate Triferuloyl spermidine (m/z 147.0446, RT 6.72 min) was detected, indicating the potential for production of phenolic acids with antioxidant and pathogen-suppressive properties.

## 4 Discussion

The *R. solanacearum* is the soil-borne bacterium that causes bacterial wilt. This bacterial disease is known to be the most detrimental to a variety of crops that are essential to agriculture, including eggplant, tomatoes, peppers and potatoes (Cai et al., 2021). *F. oxysporum*, *R. solani* and *S. rolfsii* can also rapidly spread and compromise crop yield (Zape et al., 2014). Agricultural sustainability can be ensured by the use of biocontrol agents as a mitigation strategy. The application of biological control agents has grown in contrast to chemical control techniques due to their environment friendly nature. The main aim of pathogen control to protect plant health is to utilize different species with antagonistic effects with respect to other plant pathogens to achieve overall plant protection. In this study, isolation of endophyte from the roots of the tomato plant and identification were done on the basis of 16 s RNA gene sequencing as well as whole genome-based taxonomy assignment. *S. maltophilia* 3A was identified and showed 99.89, and 99.02% similarity with *S. maltophilia* and *S. maltophilia* SG.Y2 strain based on BLAST, and GTDBtk analysis, respectively. This study reported a high-quality genome assembly of *S. maltophilia* 3A with 99.82% genome completeness and only one missing gene. Numerous studies reveal *S. maltophilia* as a potent biocontrol agent (Fariman et al., 2022). Numerous studies reveal *S. maltophilia* as a potent biocontrol agent (Fariman et al., 2022). Considering a prior study by Sultana and Hossain (2022), their study’s dual-culture experiments revealed that *S. maltophilia* strain PPB3 exhibited approximately 88% inhibition of *S. rolfsii,* while *Bacillus subtilis* strain PPB9 demonstrated approximately 71% suppression. Results from their investigation conclude the potential application of these bacteria as bio-control substitute for tomato Southern blight. Strain 3A showed strong antifungal activity in dual-culture assays. The significant inhibition was found against *S. rolfsii*. Further investigation of strain 3A’s antagonistic potential against nine isolates of *R. solanacearum* revealed that strain RS3 had the highest inhibition rate. Recently, it has been reported that numerous bacterial strains have been effectively employed to boost plant growth and inhibit diseases caused by the pathogen *S. rolfsii, including Rhizobium, Ensifer, Pseudomonas, Bacillus, Ochrobacturm, Phyllobacterium, Herbspirillum, Shinella, Alcaligenes, Azospirillum, Azotobacter, Mesorhizobium, Devosia, Clostridium, Enterobacter,* and *Klebsiella* (Riaz et al., 2022; Kumari et al., 2021). A study conducted by Elhalag et al. (2016) reported the biocontrol activity of *S. maltophilia against R. solanacearum* and showed systemic resistance under different optimized conditions.

The results of the study with different cell fractions (Figure 2) showed that the whole cells contains both bacteriostatic and bactericidal type of metabolites which does not allowed the cells to reappear from suppression zone. However, the extracellular extract was having more of bacteriostatic sub-cidal metabolites as in the later part of incubation, the high polysaccharide producing cells appeared from suppression zone, showing incomplete killing. As the bacteriostatic pressure at the beginning allows the *Ralstonia solanacearum* cells to induce the production of cells with high exopolysaccharide production, the effects of exopolysaccharide suppression might not be found as seen from sonicated extract. The absence of cidal type of metabolites, the sonicated fraction could not induce pressure on the colonies, and thus the small fraction of metabolites which could suppress the exopolysaccharide production could work well. Showing the possibility that in sonication only those metabolites could come in the cell free extract, which were having suppressive effects to the exopolysaccharide production.

The colonization capability of *S. maltophilia* 3A was further assessed on tomato roots. Extensive colonization with intense green fluorescence, as observed in under CLSM, indicates live bacterial cells distributed along the root epidermis and in intercellular spaces. Various plant growth-promoting microbes showed root colonization under the plant defence system using confocal microscopy. In support of this study, results of the investigation conducted by Rufián et al. (2018) reveal the extensive colonization by bacterial strains as dual or mono culture, which can be seen in the spaces between cells that surround mesophyll cells, and was distinguished by the red fluorescence of their chloroplasts. Confocal laser scanning and scanning electron microscopy (CLSM) of primary roots colonized by *P. fluorescens* A6RI, 7 days after inoculation, showed numerous bacterial cells evenly distributed in the zone and clustered in a study based on understanding the colonization pattern of primary tomato roots by *P. fluorescens* A6RI reported by Gamalero et al. (2004).

Secondary metabolites, including phenolic compounds, are other biologically active compounds that may be responsible for the strong antagonistic activity as demonstrated by FTIR analysis, which showed complex functional group analysis. The FTIR spectra of the crude chloroform extract of *S. maltophilia* 3A identified multiple distinctive absorption peaks, showing various chemical groups, including phenolic compounds, alkanes, esters, carboxylic acids and hydroxyl groups. Phenolic acids have shown effectiveness in combating serious fungal diseases, especially as strains of *Candida albicans*, which can also infect human beings Zabka and Pavela (2013). Metabolites identified in a study by Puopolo et al. (2013), as phenazine-1-carboxylic acid (PCA) and 2-hydroxyphenazine (2-OH P), which are carboxyl group derivatives, are a structural feature important for the antifungal activity. These groups can play a potential role in antimicrobial activity.

This study also explored the metabolomic profiling, which reveals the distinguished range of metabolites in *S. maltophilia* 3A. The future prospects of metabolomics approaches in sustainable agriculture appear hopeful since they offer an efficient means for quantitatively screening, comparing, and validating the metabolites produced by bacteria. A total of 1,612 metabolites were detected using the sophisticated LC-HRMS approach. Metabolite characterization demonstrated the presence of polyketides, alkaloids, fatty acids, and flavonoid-like compounds. The majority of plant tissues contain high concentrations of flavonoids, which have been shown to serve crucial functions in defense, development, and hormone control in plants. However, it is yet unknown how functionally active flavonoids are in humans (Tauchen et al., 2020). The protective mechanisms indicated by polyphenols consist of stimulation of apoptosis, detoxification of xenobiotics, immune system stimulation, and anti-inflammatory properties. Polyphenols are known to have the capacity to regulate the biological adaptation by sustaining the immune system and shielding cells from oxidative damage (Niedzwiecki et al., 2016). Our Metabolomics results also revealed a diverse array of secondary metabolites in *S. maltophilia* 3A. The most abundant metabolite classes identified were organonitrogen compounds, notably alkaloids and derivatives, organic acids and derivatives, benzenoids, lipids and lipid-like molecules, and organoheterocyclic compounds. Other significant classes included phenylpropanoids and polyketides, organosulfur compounds, and polyketides, highlighting a wide range of their functional relevance. Plant secondary metabolites, including phenolic acids, lignins, monolignols, phenylpropanoids, flavonoids, and coumarins, possess significance for both biotic and abiotic stress responses in addition to their relationships with the external environment. The antifungal properties of phenylpropanoids and flavonoids are effective against *Alternaria alternata, R. solani,* and *F. oxysporum* (Kumar et al., 2023). Isoquinoline Alkaloids and Indole or Benzopyrole Alkaloids have the anti-microbial and anti-tumor mitigating activities that these alkaloid-derived compounds significantly show (Mohiuddin, 2019). Reported by Shang et al. (2022), two important groups of N-based heterocyclic chemicals, quinoline and quinazoline alkaloids, have been identified from natural sources and have significant bioactivities in the vast majority of them and their substituted analogues. We also analyse the diverse functional roles of metabolites identified from the crude extract of *S. maltophilia* 3A using compound-function interaction network. Some key antibiotic-related metabolites, such as myxochelin, clavastatin, and rubratoxin tetra-acetate, clustered around the “Antibiotic” node, indicating a strong potential for microbial antagonism. Thus, our observations lead to support that these compounds can significantly affect the plant-microbe interactions and their bioactive compounds have high value for future applications.

The Dot plot analysis of enriched metabolite sets showed “organonitrogen compounds” (especially organoheterocyclic compounds) and “phenylpropanoids/polyketides,” which have the lowest *p*-values (*p* < 0.001) are the most significantly enriched pathways with high enrichment ratios. Among the pathways explored in our study using KEGG-based pathway enrichment assessment, the glycerophospholipid pathway, phenylpropanoid pathways, indole alkaloid pathway, and diterpenoid pathway also showed significant contribution. In accordance with our results, Guinan et al. (2018) claimed that secondary bile acids might be able to restrict the metabolic processes of the prominent fungal pathogen, *C. albicans*. The endophytic bacteria with significant inhibitory action were identified by Cao et al. (2021), who also used metabolomics to explain potential processes for resistance acquisition. Their findings demonstrated that mdj-36 exhibited the highest level of *F. oxysporum* inhibition during *in vitro* conditions. Glycerophospholipid components such as phosphatidic acid and lysophosphatidyl ethanolamine are critical for host microbe interaction, biofilm formation and membrane fluidity (Li et al., 2022). However, inter-species signalling and antimicrobial activity are predominantly linked to the production of indole alkaloids (Odularu et al., 2022). According to Nicholson and Hammerschmidt (1992), phenylpropanoids are known to help in plant defence. So, finding compounds involved in phenylpropanoid pathways may suggest that host plants could become more resistant to disease.

This study also explores the biosynthetic gene clusters that are involved in the production of secondary metabolites or antimicrobial peptides in *S. maltophilia* 3A. To identify the gene clusters, we used antiSMASH software for prediction and NCBI BLAST for comparative analysis of the secondary metabolite gene clusters with functionally known clusters. So, mining of *S. maltophilia* 3A genome, suggested its ability to the production of diverse antibiotics and antimicrobial peptides belonging to different classes of biosynthetic gene clusters and structural compounds such as Arylpolyene, Terpene-precursor, ribosomally synthesized and post-translationally modified peptides (RiPP), Lassopeptides, Polyketides (PKS), non-ribosomal peptide (NRP) -metallophore and NRPS. Among the identified gene clusters, several showed significantly low similarities with known clusters, including clusters of regions 2.1, 3.1 and 5.1. These findings predicted that this endophyte has the capability to produce unique secondary metabolites and structural compounds. The presence of the arylpolyenes (APEs) biosynthetic gene cluster may enable the *S. maltophilia* 3A to produce antioxidants and protect bacteria against oxidative stress, UV stress, or host defenses (Schöner et al., 2016). The presence of similarities with the Flexirubin gene cluster is also a novel feature of this bacterium. Some gene clusters involved in the biosynthesis of secondary metabolites are predicted to be bioactive molecules of Lassopeptides, including xanthomonin, sphingonodin, subterisin, caulonodin, siamycin, which mainly contain antibiotic, signalling, or antimicrobial properties. While the highly similar region 5.2 belongs to the hybrid gene cluster of NRP-metallophore and NRPS, and most of their products are NRPS-Type I based siderophores, including 2,3-dihydroxybenzoylserine, griseobactin, benarthin/dibenarthin, mirubactin, bacillibactin, paenibactin, and myxochelin (Type I, NRPS+PKS), typically involved in iron acquisition and secondary antimicrobial functions. Among these compounds, bacillibactin is one of the most common siderophores, produced by many members of *Bacillus* spp. and functions in iron scavenging (Chen et al., 2009). Therefore, we can consider that bacillibactin in *S. maltophilia* 3A may serve growth promoter by chelating with iron and reducing its absorption by pathogenic fungi (Wang et al., 2024). The presence of the NRPS gene cluster may enable *S. maltophilia* 3A to be the producer of antimicrobial metabolites and defensive compounds.

The integration of metabolomics with genomic data analysis revealed a strong association between bioactive compounds biosynthesis and identifying their BGCs. Several metabolites, plant growth promoters, signalling molecules, alkaloids, terpenoids and flavonoids were identified from the crude extracts through HRMS profiling, though not all are associated with identified gene clusters detected by antiSMASH, but to some extent, corresponding terpene precursor and polyketide synthase-encoding genes were identified in the 3A genome. AntiSMASH also identified NRPS and RiPP-like cluster-based siderophores with 100% similarity to compounds 2,3-dihydroxybenzoylserine and myxochelin A/myxochelin B of the metabolic extract. This suggests active biosynthesis of bioactive siderophore-like compounds by the bacterium. The detection of benzenoids and phenylpropanoid derivatives in the extract may be moderately correlated with arylpolyene clusters. The analysed culture extract also contained phenolic acids, flavonoids, lignins, and coumarins. Still, neither of the corresponding gene clusters was identified in the genome. One of the possible regions behind this mismatch could be the limitation of short-read sequencing. Here, the genome assembly data were generated from Illumina NovaSeq with a read length of 2 × 151 bp, which, while accurate, can lead to fragmented assemblies of the genome, so an incomplete representation of BGCs may be obtained. Incorporating long-read sequencing (such as PacBio or Oxford Nanopore) approaches or employing hybrid strategies in future studies may improve contiguity and enable more comprehensive, complete BGC reconstruction of the genome.

Therefore, this analysis may help us to deep understanding of the mechanism of *S. maltophilia* 3A antagonism and anti-microbial peptides. Our data suggest that strain 3A could help plants become resilient by causing metabolic cross-talk and acting as an immediate antagonist. These findings also support the previous evidence that these compounds can have future significance with relation to plant-microbe interaction and provide tolerance towards pathogens.

In the recent past *Stenotrophomonas maltophilia* has been reported as a PGPR and biocontrol agent by demonstrating its role in suppressing phytopathogens and promoting plant growth and development mediated by the production of phytoharmones, enzymes, and volatile organic compounds (Alijani et al., 2020; Sharma et al., 2024). However, *S. maltophilia* is also recognized as an opportunistic human pathogen, raising legitimate biosafety concerns regarding its agricultural application. Importantly, the ecological behavior and pathogenic potential of this species are highly strain-dependent. It is important to note that pathogenicity is not an inherent or universal trait of all strains, being opportunistic its context-dependent phenomenon. Such pathogens typically cause infection only under specific conditions mainly under immune-compromised hosts, in hospital environments, technically when normal microbiota barriers are disrupted. In environmental situation, including soil and plant-associated microbiomes, *S. maltophilia* is widely distributed and often functions as a commensal or beneficial organism rather than a pathogen. Moreover, its interactions within the rhizosphere microbiome have an influence over efficacy in ecological outcomes, including interference with other beneficial microorganisms (Peng et al., 2025). However, to overrule ambiguity, detailed genomic and phenotypic characterization, including screening for virulence determinants and antibiotic resistance, would comply with biosafety regulations and thorough environmental risk assessment before field application.

## 5 Conclusion and future prospects

The *S. maltophilia* 3A exhibits strong biocontrol potential, primarily through the production of antimicrobial metabolites that directly suppress plant pathogens. Metabolomic analysis revealed several bioactive compounds with known antibacterial and antifungal properties, supporting their role in pathogen inhibition. These findings highlight the potential for developing metabolite-based formulations as effective, eco-friendly alternatives to conventional agrochemicals. The proposed gene clusters helped identify the bioactive metabolites and antimicrobial potential of *S. maltophilia* 3A. Future work should focus on understanding their modes of action, and enhancing their production through multi-omics and metabolic engineering approaches. This study provides a foundation for advancing endophytic metabolites as targeted biocontrol solutions in sustainable agriculture.

## Data Availability

The datasets presented in this study can be found in online repositories. The names of the repository/repositories and accession number(s) can be found in the article/Supplementary material.
