# Supplementary material for: Untargeted metabolomic profiling and genome mining of endophytic Stenotrophomonas maltophilia strain 3A reveal a rich source of bioactive secondary metabolites
Source: Front Microbiol. 2026 May 12;17:1792452. doi: 10.3389/fmicb.2026.1792452 (PMC13201425; doi:10.3389/fmicb.2026.1792452)
Supplement: Supplementary file 2 [file Table_2.docx]

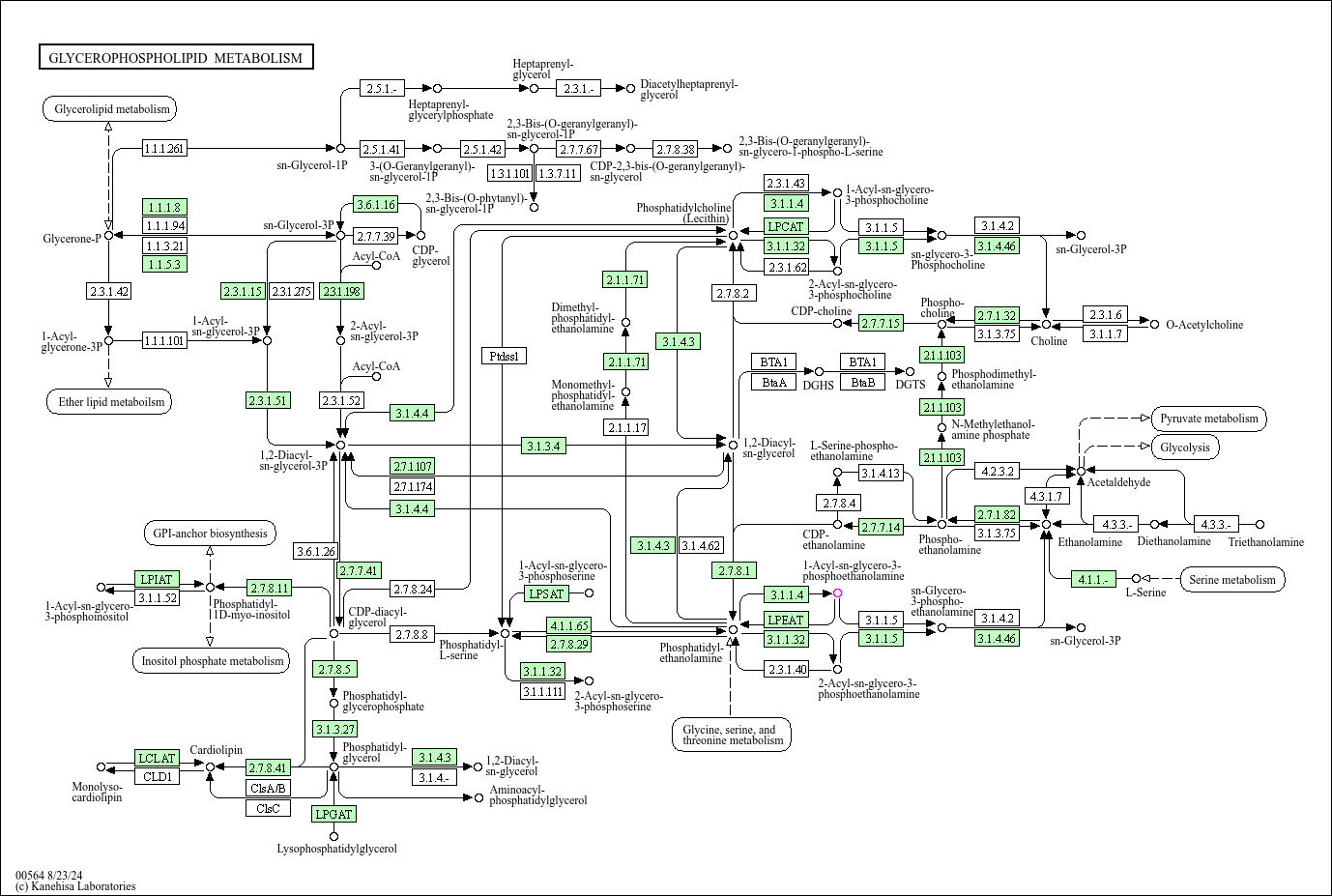


Supplementary Figure S1: Glycerophospholipid biosynthesis pathway generated using KEGG database in MetaboAnalyst 5.0 highlighting its significant contributions in *Stenotrophomonas maltophilia* 3A.


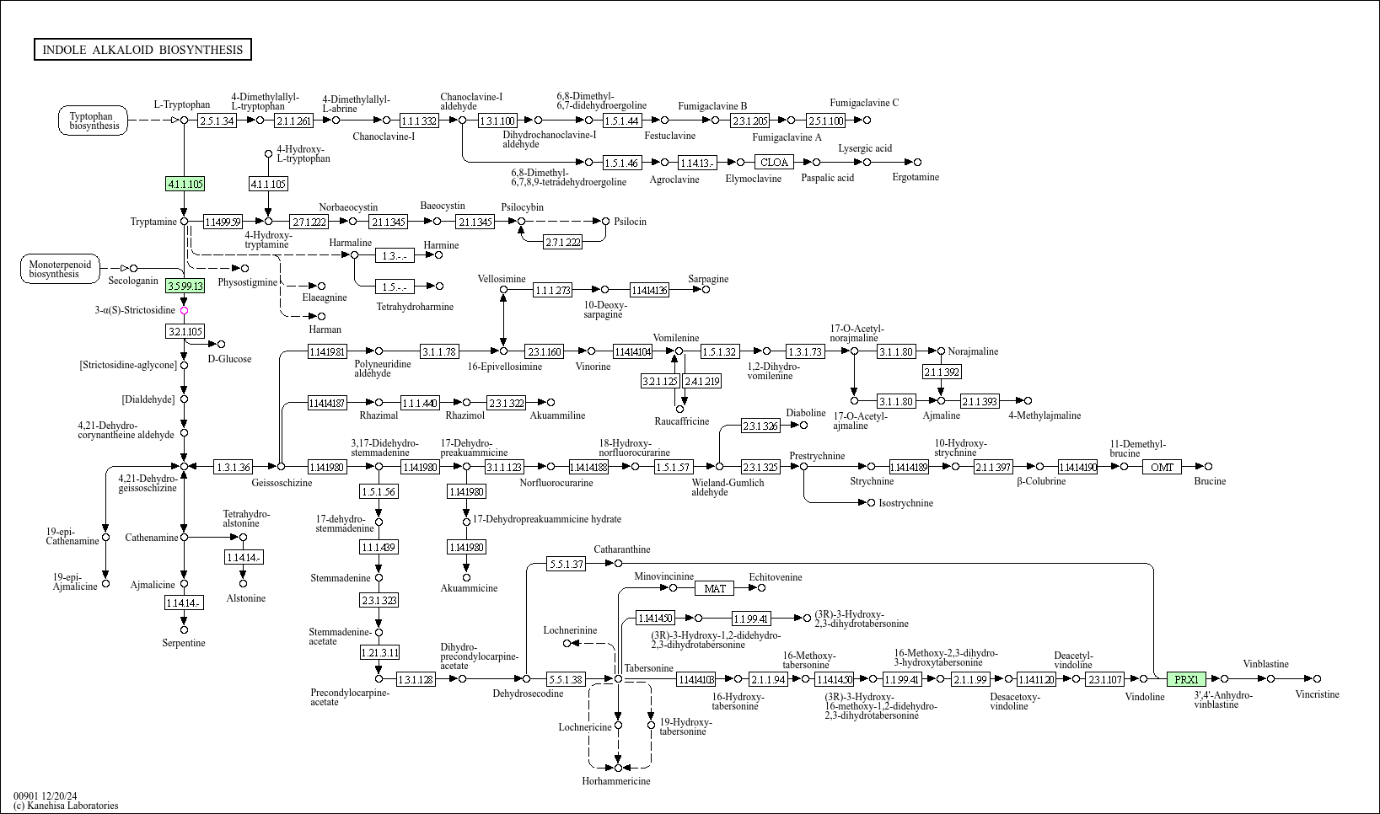


Supplementary Figure S2: Indole alkaloid biosynthesis pathway generated using KEGG database in MetaboAnalyst 5.0 highlighting its significant contributions in *Stenotrophomonas maltophilia* 3A.


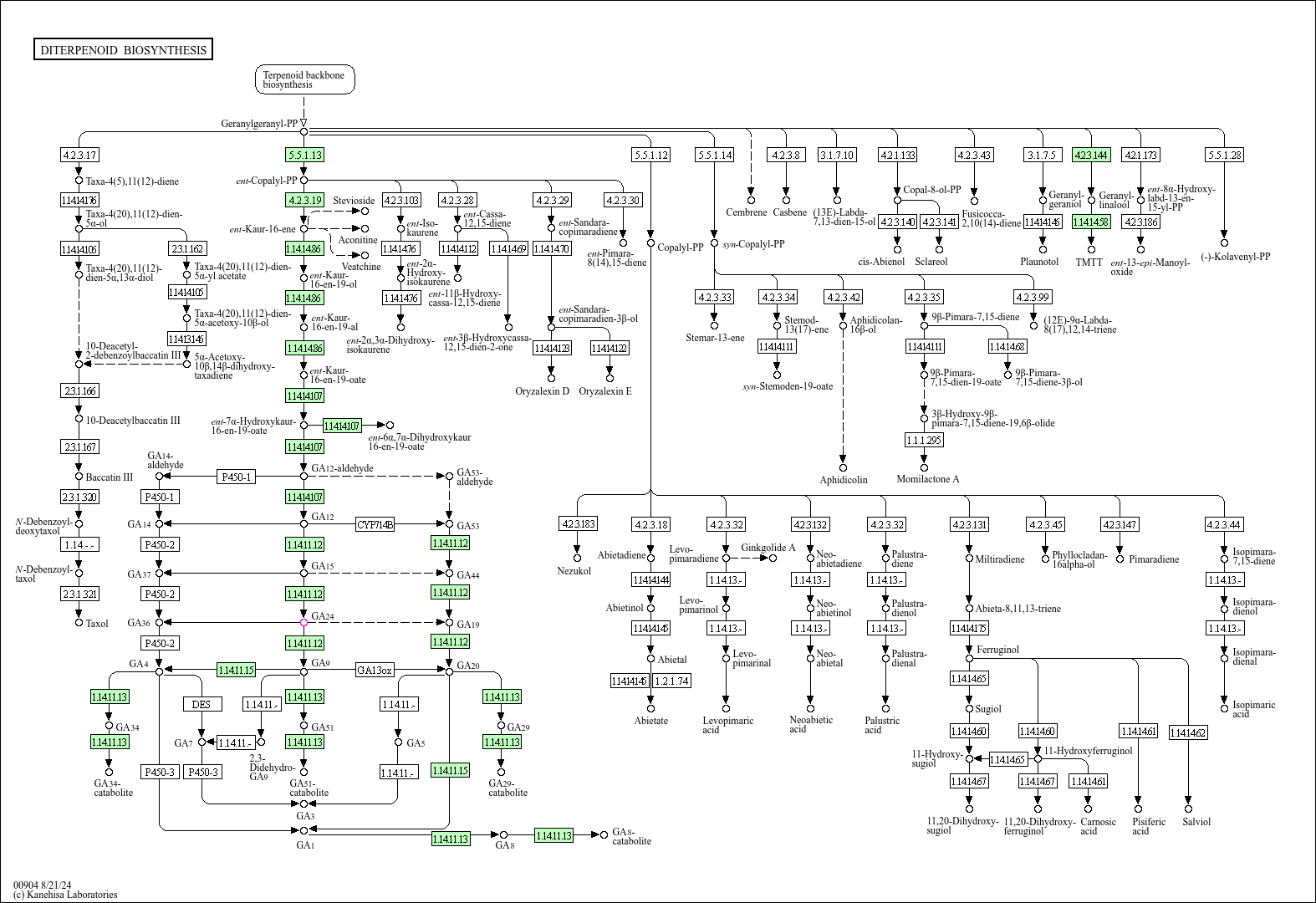


Supplementary Figure S3: Diterpenoid biosynthesis pathway generated using KEGG database in MetaboAnalyst 5.0 highlighting its significant contributions in *Stenotrophomonas maltophilia* 3A.


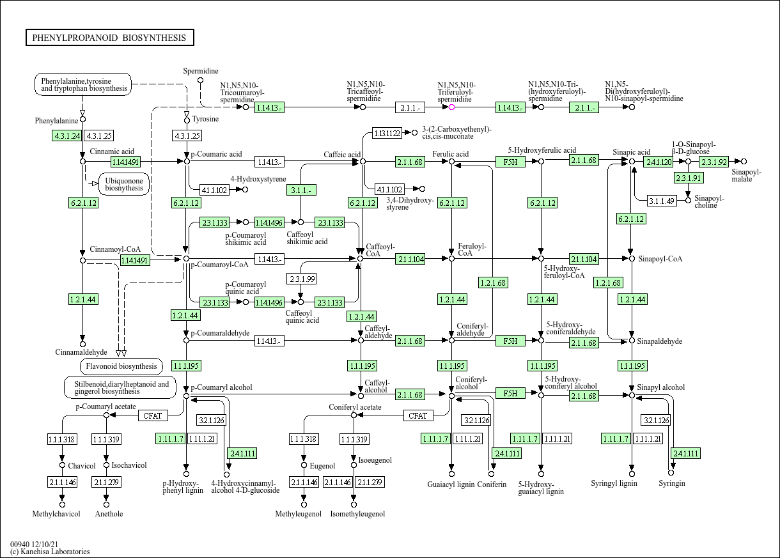


Supplementary Figure S4: Phenylpropanoid biosynthesis pathway generated using KEGG database in MetaboAnalyst 5.0 highlighting its significant contributions in *Stenotrophomonas maltophilia* 3A
